# Constitutional Mosaic Epimutations - a hidden cause of cancer?

**DOI:** 10.15698/cst2019.04.183

**Published:** 2019-03-22

**Authors:** Per E. Lønning, Hans P. Eikesdal, Inger M. Løes, Stian Knappskog

**Affiliations:** 1 K.G.Jebsen Center for Genome Directed Cancer Therapy, Department of Clinical Science, University of Bergen, Norway.; 2 Department of Oncology, Haukeland University Hospital, Bergen, Norway.

**Keywords:** epimutations, promoter methylation, cancer risk, BRCA1, MLH1

## Abstract

Silencing of tumor suppressor genes by promoter hypermethylation is a key mechanism to facilitate cancer progression in many malignancies. While promoter hypermethylation can occur at later stages of the carcinogenesis process, constitutional methylation of key tumor suppressors may be an initiating event whereby cancer is started. Constitutional *BRCA1* methylation due to *cis*-acting germline genetic variants is associated with a high risk of breast and ovarian cancer. However, this seems to be a rare event, restricted to a very limited number of families. In contrast, mosaic constitutional *BRCA1* methylation is detected in 4-7% of newborn females without germline *BRCA1* mutations. While the cause of such methylation is poorly understood, mosaic normal tissue *BRCA1* methylation is associated with a 2-3 fold increased risk of high-grade serous ovarian cancer (HGSOC). As such, *BRCA1* methylation may be the cause of a significant number of ovarian cancers. Given the molecular similarities between HGSOC and basal-like breast cancer, the findings with respect to HGSOC suggest that constitutional *BRCA1* methylation could be a risk factor for basal-like breast cancer as well. Similar to *BRCA1*, some specific germline variants in *MLH1* and *MSH2* are associated with promoter methylation and a high risk of colorectal cancers in rare hereditary cases of the disease. However, as many as 15% of all colorectal cancers are of the microsatellite instability (MSI) “high” subtype, in which commonly the tumors harbor *MLH1* hypermethylation. Constitutional mosaic methylation of *MLH1* in normal tissues has been detected but not formally evaluated as a potential risk factor for incidental colorectal cancers. However, the findings with respect to *BRCA1* in breast and ovarian cancer raises the question whether mosaic *MLH1* methylation is a risk factor for MSI positive colorectal cancer as well. As for *MGMT*, a promoter variant is associated with elevated methylation across a panel of solid cancers, and *MGMT* promoter methylation may contribute to an elevated cancer risk in several of these malignancies. We hypothesize that constitutional mosaic promoter methylation of crucial tumor suppressors may trigger certain types of cancer, similar to germline mutations inactivating the same particular genes. Such constitutional methylation events may be a spark to ignite cancer development, and if associated with a significant cancer risk, screening for such epigenetic alterations could be part of cancer prevention programs to reduce cancer mortality in the future.

## INTRODUCTION

Malignant tumors are thought to arise through a sequence of genetic disturbances [1]. In some cancers, like colorectal carcinomas, the sequence of key genomic events in general follows a common order [2], while in other cancer types, like breast cancer, the sequence of events in carcinogenesis seems to occur at random [3, 4].

The identification of genomic aberrations predisposing to cancer have added substantially to our understanding of cancer-inducing events. Importantly, the finding that germline mutations in genes like *BRCA1/2*, *TP53*, *RB1*, *CDKN2A* and others are associated with an elevated risk of certain cancers indicates that mutations in these genes may act as the initial events in malignant transformation in sporadic cancers as well [5].

**Figure 1:**
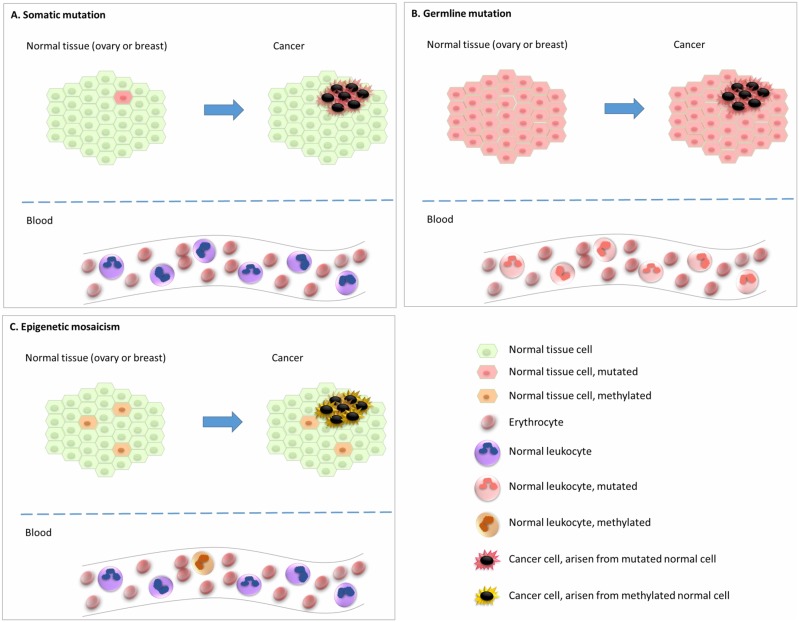
FIGURE 1: Early events underlying carcinogenesis. **(A)** Cancer arising from normal cells subject to a somatic driver mutation as the initial event, with subsequent alterations leading to malignant transformation. In such cases the initial event will not be detected in white blood cells (WBC). **(B)** Cancer arising from normal cells harbouring a germline driver mutation, acting as the initial event, with subsequent alterations leading to malignant transformation. In such cases the “initial event” will be detectable in all WBCs. **(C)** Cancer arising from a minority of normal cells with a key tumour suppressor methylated from early embryonic life (epigenetic mosaicism). This methylation may act as the initial event, with subsequent alterations leading to malignant transformation. In such cases the initial event / methylation will be detectable also as mosaicism in WBC.

In addition to gene mutations and rearrangements, somatic epigenetic alterations, i.e. *epimutations*, affecting gene expression levels can play a pivotal role during carcinogenesis [6]. Further, recent findings have indicated that underlying epimutations of certain genes in the normal tissue are associated with an elevated risk of particular cancer subtypes. This indicates that an epigenetic, and not genetic, event may be the initial step in the carcinogenesis process for these particular cancers (**Figure 1**). Of notice, epimutations that are widely distributed across normal tissues, predisposing to disease, are defined as constitutional epimutations [7–10]. By definition, constitutional epimutations involve tissue derived from all the three germ layers [9]. Thus, in order to distinguish constitutional methylation from gene methylations acquired during lifetime, it is important to assess methylation status of the gene in question across tissues derived from more than one of the germ layers (endoderm, ectoderm, as well as mesoderm).

Studies of normal tissues show that epimutations which affect only a fraction of the alleles may still mediate an increased cancer risk. On the other hand, in a malignant tumor arising from a precursor cell carrying an epimutation, we may expect all cells in the resulting tumor to harbour the epigenetic event if it is essential to cancer progression. While epigenetic gene silencing involves several types of modifications, including DNA methylation, histone modifications and RNA interference, in this review we will focus on promoter CpG methylation, the only mechanism that has been explored with respect to cancer predisposition in human studies so far.

Evidence linking epimutations to cancer risk has been reported for a limited number of genes. For these particular genes, we will discuss the evidence indicating normal tissue epimutations to infer an elevated cancer risk. Additionally, we will discuss the potential clinical and biological importance of these epimutations with respect to how they may mediate the phenotype of particular cancers. Of notice, if epimutations act as the initial trigger events, we expect such malignancies in general to mirror the phenotype of cancer in the same organ developing due to germline mutations in the same gene. This is in contrast to sporadic cancers where one would expect a more diverse phenotype based on heterogenous genomic events triggering cancer initiation.

The Lynch syndrome [11] or hereditary non-polyposis colorectal cancer (HNPCC), is an autosomal dominant genetic condition characterized by an elevated risk of colorectal cancer, with a preponderance for proximal/right-sided colon cancers, as well as an elevated risk of endometrial cancer. The syndrome is caused by defects in DNA mismatch repair due to germline mutations in either *MLH1* or *MSH2*, or, more rarely, in the *MSH6*, *PMS1* or *PMS2* genes [12]. Additionally, recent findings have revealed a moderately increased risk of various other solid malignancies as part of the Lynch syndrome, affecting the stomach, small intestine, pancreas, hepatobiliary and upper urinary tract, brain, ovary or breast [11].

BOX 1 SCIENTIFIC QUESTIONS THAT NEED TO BE ADDRESSED

**1.** *MLH1* and *MGMT*, as well as other genes which are methylated in cancer tissues, should be assessed with respect to mosaic methylation in WBC or other types of normal tissue from healthy individuals. If they are methylated in a distinct fraction of the population (using *BRCA1* as “standard”; > 4%), one should assess the OR for individuals harbouring constitutional methylation to develop the same type of cancer.

**2.** To what extent is constitutional mosaic promoter methylation affecting key tumour suppressors, apart from *BRCA1*?

**3.** What is the cause of neonatal promoter methylation of *BRCA1*?

**4.** We do not know whether promoter methylation of genes like *BRCA1* remains static during life or fluctuates in a dynamic state. To follow individuals over decades collecting regular blood samples may be difficult, but efforts should be made to address this question.

Due to the mismatch repair gene defects, malignant tumors associated with Lynch syndrome are characterized by a microsatellite-instability (MSI) phenotype [13]. While the Lynch syndrome accounts for only 2-3% of all colorectal cancers [14, 15], up to 15% of all colorectal cancers are defined as MSI “high”, where other underlying mechanisms must be at play [16]. Interestingly, the majority of these MSI "high" tumors reveal somatic hypermethylation of the *MLH1* promoter region [17–19]. Moreover, all malignant tumors with MSI are characterized by a similar phenotype, regardless of which of the mismatch genes that are mutated - this includes MSI “high” cancers due to *MLH1* inactivation; whether it is by germline mutations or epigenetic silencing [20]. Compared to other colorectal cancers, MSI high tumors are characterized by a high mutation burden, and they also seem to draw a substantial benefit from immunotherapy [21, 22].

Characteristics such as MSI and a high mutation load in malignancies from patients with Lynch syndrome or spontaneous cancers harboring *MLH1* promoter methylation indicate that epimutations, as well as somatic mutations affecting *MLH1,* are early events during malignant transformation [23]. Alternatively, for tumors harbouring *MLH1* hypermethylation, if the high mutational load occurs at a later stage it must have been selected for through a profound “selective sweep” [24]. Interestingly, Miyakura *et al.* [19], in addition to analyzing for *MLH1* methylation in the tumor tissue, examined *MLH1* methylation status in the matching normal colon mucosa, detecting partial *MLH1* promoter methylation in one third of the patients. Although no firm conclusions can be drawn from this finding, one may speculate that in a subset of patients the carcinogenesis process may have started by methylation of normal colon mucosal cells.

## CONSTITUTIONAL *MLH1* METHYLATION AND COLORECTAL CANCER RISK

As defined by Hitchins and colleagues [25, 26], epimutations may be separated into two major groups; primary epimutations (or promoter methylation) where no DNA alterations are detected, and secondary epimutations, occurring in concert with (and caused by) a local *cis*-acting DNA alteration. Following the findings by Kane and colleagues [27] demonstrating *MLH1* promoter methylation in colorectal tumor tissue, and the report of Gazzoli and colleagues in 2002 [28] demonstrating white blood cell (WBC) DNA methylation of the *MLH1* promoter in a young man diagnosed with an MSI positive colon cancer, *MLH1* methylation has been detected in circulating leucocytes (WBC) of a subset of patients with sporadic MSI positive colorectal cancers. In some cases, such findings have also been made in probands without a cancer diagnosis. However, less than 50 individuals have been reported in the literature with concurrent colorectal cancer and constitutional (normal tissue) *MLH1* methylation so far [29–42]. As for studies reporting the fraction of methylated alleles in the blood of these individuals, this has been in the range of 20-50% [39], with a monoallelic pattern.

In 2011, Hitchins and colleagues identified a haplotype harbouring tandem nucleotide substitutions, where a c.-27C>A variant was the likely cause of *MLH1* methylation and cancer diagnosis across a family with Lynch syndrome [38]. Additionally, in a few cases, methylation has been detected in concert with larger genomic rearrangements of the *MLH1* gene [35, 37]. Apart from these individuals, the potential pathogenic contribution of genomic rearrangements to *MLH1* methylation remains an open question.

Contrasting the “high-level” methylation associated with colorectal cancer mentioned above, low level mosaic WBC methylation of *MLH1* in patients with colorectal cancer has also been reported [29, 39]. The potential contribution of such low level methylation in *MLH1* to colorectal cancer risk remains to be formally assessed. Anecdotally, Sloane and colleagues [43] reported a young male diagnosed with colorectal cancer to harbour constitutional methylation in about 50% of his alleles, while his mother revealed mosaic *MLH1* methylation in less than 5% of the alleles. Interestingly, among retinoblastoma patients diagnosed with germline *RB1* mutations, in some cases an unaffected parent carried the same mutation at low frequency in her/his WBCs [44, 45]. Mosaic gene methylation as a cancer risk factor will be discussed further as part of reviewing *BRCA1* methylation data below.

## MSH2

While *MSH2* methylation was detected in a small subset of colorectal cancers, most importantly it appeared only in subfractions of the malignant cells, with no correlation to gene expression level or MSI status [46]. Constitutional methylation of *MSH2* is a rare event, first described by Chan *et al.* [47] in 2006. In a subsequent study [48], the same family was further characterized together with an additional set of nine Dutch and Chinese families. In summary, patients in these families all revealed loss of MSH2 staining by immunohistochemistry (IHC) and hypermethylation of the *MSH2* promoter within the colorectal cancers, as well as methylation of the *MSH2* promoter across various normal tissues, although to a variable extent. Importantly, all patients carried a deletion in a gene upstream of *MSH2*, namely *TACSTD1* (encoding Ep-CAM). This deletion resulted in *MSH2* promoter methylation and reduced *MSH2* transcription in the colon mucosa and subsequent colorectal cancer cells. This finding was confirmed by Niessen and colleagues in another three independent individuals carrying the Lynch syndrome [42].

## O-6-METHYLGUANINE-DNA METHYLTRANSFERASE (*MGMT*)

*MGMT* is downregulated by promoter methylation in various types of cancers [49–57]. Subsequent loss of methylation and re-elevated expression of *MGMT* has been associated with resistance towards alkylating agents like temozolomide and cyclophosphamide [49, 50, 55, 58–60]. While germline mutations in *MGMT* have not been detected so far, the T-allele of the single nucleotide polymorphism (SNP) rs16906252, located in the first exon of *MGMT*, close to the transcription start site, has been associated with elevated promoter methylation across a panel of solid malignancies [51, 53, 54, 61, 62]. Mirroring findings for *MLH1* (see above), Shen and colleagues [52] detected *MGMT* methylation not only in cancer tissue, but also in normal colon mucosa located 10 cm from the tumor borders. More recently, mosaic *MGMT* methylation (up to 10% of the alleles) associated with the rs16906252 SNP T-allele has also been detected in WBC [63]. In a large study of germline genotypes (WBC) including a validation cohort, Kuroiwa-Trzmielina and colleagues found the rs16906252 T-allele to be associated with an odds ratio (OR) of 3-4 for harbouring *MGMT* promoter methylation within a colorectal cancer [56]. In addition, one smaller study found a moderate but significant association between the rs16906252 T-allele of *MGMT* and glioblastoma risk [61]. Taken together, these studies indicate that the rs16906252 SNP may affect the risk of different cancers by causing increased *MGMT* promoter methylation.

## *BRCA1* AND *BRCA2* EPIMUTATIONS IN BREAST AND OVARIAN CANCER TISSUE

Women carrying germline pathogenic mutations in *BRCA1* and *BRCA2* are at high risk of developing breast as well as ovarian cancer [64–67]. Notably, germline mutations in *BRCA1/2* have also been linked to an elevated risk of cancer of the prostate and pancreas [68, 69], and germline *BRCA2* mutations to a moderately increased risk of several other malignancies [70, 71]. With respect to the current review, evidence linking *BRCA1/2* methylation to cancer risk has so far only been collected from patients with breast and ovarian cancer.

*BRCA1* and *BRCA2* both participate in homologous DNA repair. *BRCA2* is part of the Fanconi complex (*FANCD1*), whereas *BRCA1* has a critical role as a downstream executor of this complex [72, 73]. However, the breast cancer phenotypes linked to deficiencies in these two gene varies. As for breast cancers arising in *BRCA1* mutation carriers, >80% belongs to the so-called “basal-like” subtype [74], accounting for the majority of triple negative breast cancers [75]. This contrasts spontaneous breast cancers where triple negative tumors account for approximately 15% [76, 77]. On the other hand, tumors arising in *BRCA2* mutation carriers reveal a phenotype distribution mirroring spontaneous breast cancers [78]. Among basal-like breast cancers, 10-25% are associated with a germline *BRCA1* mutation. This rather wide range is due to differences in ethnicity and age distribution at cancer diagnosis in different studies [79, 80].

While somatic *BRCA*1/2 mutations in breast cancer previously were thought of as rare, compared to germline mutations, contemporary evidence indicates that one third of *BRCA* mutations have a somatic origin [4, 81–86]. Moreover, mutations of *BRCA1/2* as well as other crucial DNA repair genes inflict homologous repair deficiency (HRD), which is associated with distinct gene mutation signatures, including copy number variations [84]. Thus, different mutational signatures aiming at predicting HRD have been generated [87–89]. Applying such a signature assessment to breast cancers have indicated that HRD may characterize as many as 20% of all cases [87]. The biological and clinical relevance of such signatures are underlined by merging evidence validating their role in predicting sensitivity towards treatment with PARP (Poly-ADP-Ribose-Polymerase) inhibitors as well as certain chemotherapy regimens, resembling what may be seen for patients harbouring germline *BRCA1/2* mutations [83, 90–94]. The reason for the homologous repair defect in most of these tumors remains unknown, but *BRCA1* promoter methylation has been reported in 30-35% of all triple negative breast cancers with germline *BRCA1/2* wild-type status, in particular among tumors of the basal-like subtype [95]. Further, *BRCA1* promoter methylation has been associated with transcriptional downregulation of *BRCA1* [96–99]. The incidence of *BRCA1* methylation is lower (5-25%) among breast cancers that are not basal-like [79, 100–107], consistent with the subtype skewness seen for *BRCA1* mutation carriers [108, 109]. Further, conflicting evidence has indicated similarities with respect to drug sensitivity and outcome between individuals with breast cancers harbouring *BRCA1* mutations and those with promoter methylation [83, 101, 110–114]. In spontaneous breast cancer, the *BRCA2* methylation frequencies vary between 0 and 12% [105, 107]. Notably, promoter methylation of *PALB2,* another gene in the Fanconi complex, has been detected in a small number of spontaneous breast cancers as well [115].

The reported *BRCA1/2* methylation frequencies vary substantially between different clinical studies. Similar to variation in the incidence of *BRCA1/2* germline mutations this could be due to ethnic variations or the age distribution in the patient cohort undergoing analysis. However, the reported frequency differences are most likely due to methodological differences.

Approximately 50% of patients diagnosed with a high-grade serous ovarian cancer seem to harbour homologous repair deficiencies in the tumor tissue [116]. High-grade serous ovarian cancer is the cancer subtype for which germline *BRCA1/2* mutations are detected at the highest frequency with 8-15% carrying a *BRCA1* and 4-8% a *BRCA2* mutation [98, 117–119]. In addition, The Cancer Genome Atlas [98] reported somatic *BRCA1/2* mutations in a small number of cases. *BRCA1* methylation is detected in 9-15% of spontaneous cases of serous ovarian cancer, but does not seem to occur in concert with germline mutations [97, 120, 121]. Notably, ovarian cancer tissue methylation for the *BRCA1* promoter, similar to germline *BRCA1* mutation status, was associated with the high-grade serous cancer subtype and young age at diagnosis [122, 123]. In contrast to the frequencies reported in *BRCA1*, methylation of *BRCA2* occurs in <1% of ovarian cancers [124–126]. In germline mutation carriers, no *BRCA2* methylated ovarian cancer has been detected so far [127].

While most breast cancers carrying *BRCA1* mutations undergo loss-of-heterozygosity (LOH) of their wild-type allele as their second hit, *BRCA1* and *BRCA2* promoter methylation have also been detected in some tumors without LOH [104, 127, 128]. However, the methylation profile varies across individual CpG nucleotides [107], and a recent study found LOH for *BRCA1* as well as *BRCA2* to occur in concert with promoter methylation of the same gene in different subclones of the same tumor [129].

In patients with ovarian cancers that are wild-type for *BRCA1/2*, *BRCA1* promoter methylation predicted better outcome to platinum-taxane based therapy [130] as well as PARP inhibition, as compared to patients without such methylation, thus mirroring findings in patients harbouring germline mutations [131–133]. Contrasting this are results from the recently published TnT trial, where patients with triple negative metastatic breast cancer and tumor *BRCA1* methylation did not respond any better to platinum chemotherapy than those without such epimutations [83]. However, methylation analyses were performed on archival tumor tissue extracted at the time of the first breast cancer diagnosis, which could have skewed the results, compared to an analysis of cancer biopsies taken at screening before entering the trial, but after previous exposure to adjuvant chemotherapy.

## NORMAL TISSUE *BRCA1/2* METHYLATION AND RISK OF BREAST AND OVARIAN CANCER

Few studies have assessed *BRCA2* normal tissue (or WBC) methylation status. To the best of our knowledge, no formal assessment for WBC *BRCA2* methylation with respect to breast or ovarian cancer risk has been conducted. Notably, in a recent study using a low detection limit, Peplonska and colleagues detected evidence of WBC *BRCA2* and *BRCA1* methylation in 18.3% and 21.5%, respectively, among (presumably) cancer-free participants [134]. However, their estimates are unusually high, also for *BRCA1* methylation. Until recently, *BRCA1/2* hypermethylation was not associated with increased risk of hereditary breast cancer [135, 136]. As for the studies presented, most of them contained a limited number of patients, raising the question of potential publication bias (negative studies may not have been reported). Also, methylation frequency within the control populations are at substantial variance across the studies due to different analytical methods and thresholds applied.

Notably, an interesting study was presented by Wong and colleagues from Dobrovic's group [108]. Analyzing a total of 255 women diagnosed with breast cancer below the age of 40 years and without germline *BRCA1/2* mutations, they detected *BRCA1* promoter methylation in WBC among 31% of patients, revealing strong morphologic characteristics (five or more individual parameters) otherwise associated with a *BRCA1* mutation. In contrast, they found peripheral blood methylation in 10% and 5% among those harboring 4 and ≤3 *BRCA1* mutation characteristics, respectively. This significantly contrasted a *BRCA1* methylation incidence of 4% among unaffected controls.

Data on constitutional *BRCA1* methylation with respect to ovarian cancer risk has in general been lacking. However, analyzing individuals wild-type for *BRCA1/2* germline mutations, Hansmann and colleages [137] identified WBC *BRCA1* methylation in 3 out of 39 patients with ovarian cancer (8%) and belonging to families with an elevated risk of ovarian and breast cancer. Among individuals recorded as *BRCA1* hypermethylated, methylation affected between 12 and 40% of the WBC alleles - thus indicating mosaic hypermethylation of this gene in the normal tissue. In the same cohort, they also identified *RAD51C* methylation in one ovarian cancer index patient.

**Figure 2:**
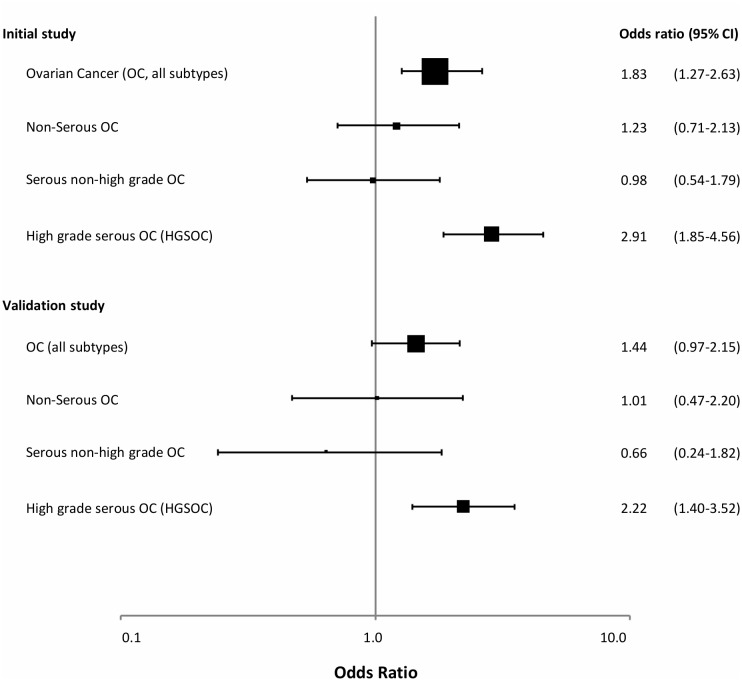
FIGURE 2: BRCA1 methylation and risk of ovarian cancer. Forest plot illustrating the odds ratio (OR) for ovarian cancer (all subtypes), non-serous, serous non-high grade and high grade serous ovarian cancer (HGSOC) related to *BRCA1* promoter methylation, derived from the initial study population and the validation cohort in the recent publication by Lønning *et al.* [131]. The odds ratios (ORs) were based on analyses of 925 cases and 1688 controls (initial study) and 607 cases and 1914 controls (validation study). Reprint of original figure, with permission from Annals of Internal Medicine.

In a recent study [138], we examined WBC *BRCA1* promoter methylation status among 1688 healthy controls and 925 patients with ovarian cancer (**Figure 2**). *BRCA1* methylation was detected by methylation-specific qPCR in 4.2% of healthy controls. While we recorded a similar methylation frequency among patients diagnosed with non-serous or low-grade serous ovarian cancers, the methylation frequency was as high as 9.6% among patients diagnosed with a high-grade serous ovarian cancer (HGSOC), revealing an OR of 2.91 (CI: 1.85 - 4.56). Our findings were confirmed in a validation cohort containing 607 patients and 1914 controls, revealing an OR for HGSOC of 2.22 (CI 1.40 – 3.52). Among patients testing positive for *BRCA1* metylation, the median percentage of methylated alleles was 4.1%, with 21% as the highest level recorded – again pointing to partial/mosaic hypermethylation of the *BRCA1* gene. Combining data from the exploratory and validation cohorts, we found an OR for HGSOC of 1.82 for individuals harbouring a methylation level below the median percentage, contrasting an OR of 4.20 for those with *BRCA1* methylation levels above the median. Finally, the OR for HGSOC associated with positive *BRCA1* methylation was highest in individuals below 50 years of age (OR 4.42). Of notice, although excluded from the formal OR assessments, we detected WBC methylation also among individuals carrying germline *BRCA1* and *BRCA2* mutations (in 1.5% and 9.0%, respectively). The biological interpretation of this potential difference between *BRCA1* and *BRCA2* mutation carriers is uncertain due to the low number of individuals analyzed.

In the same study, we examined the *BRCA1* methylation status in normal as well as ovarian cancer tissue in a subgroup of patients. Notably, we confirmed *BRCA1* methylation in various paraffin-embedded normal tissue samples from patients testing positive for WBC *BRCA1* methylation. The samples were derived from the endoderm as well as the mesoderm germ layers. While we did not have ectodermal derived samples available, it is unlikely that tissue derived from that germ layer should deviate from the other two; thus, our findings strongly indicate constitutional methylation [8, 9]. Among patients testing positive for *BRCA1* methylation in WBC, 62% were methylation positive in the tumor tissue, contrasting 12% for patients testing negative for WBC *BRCA1* methylation. These data mirror the findings by Dobrovic and colleagues in HGSOC [139]. They analyzed blood and tumor tissue from 154 patients with HGSOC and among 20 patients harbouring WBC *BRCA1* methylation, 14 of them (70%) revealed methylation of the tumor DNA as well. The finding of a 60-70% methylation frequency in tumors from individuals carrying a constitutive *BRCA1* promoter methylation is in accordance with what is expected. Assuming methylation of a small fraction of normal (including ovarian tissue) *BRCA1* alleles to be associated with an OR for HGSOC of 2.0, such a finding should indicate 50% of the cancers may arise from unmethylated cells (like in an individual not carrying any *BRCA1* methylated allele). The additional 50% of cancers would then arise from the small fraction of methylated cells. Similarly, in case of an OR of 3.0 for HGSOC, we may envision 67% of the cancers to arise from methylated cells. In our previous study, the OR in the one cohort was 2.9, and in the second cohort 2.2 [138]. Following the assumptions above, these findings correspond well with the finding of *BRCA1* tissue methylation in between 60% and 70% of the HGSOC.

In addition to the large case-control studies described above, there are also reports of special cases where *BRCA1* methylation is strongly linked to *cis*-acting genetic variants. Importantly, Evans and colleagues [140] reported constitutional *BRCA1* methylation in WBC from members of two families characterized by high incidence of breast and ovarian cancer, but testing negative for *BRCA1/2* germline mutations. Here, the methylation was associated with a 5′UTR promoter variant and about 50% of the alleles were methylated, indicating complete methylation of affected alleles. Of notice, these findings parallel the recent findings by Hitchins and colleagues described above [38] with respect to *MLH1* methylation in a colorectal cancer family. Notably, while *BRCA1* promoter variants influencing breast and ovarian cancer risk have been reported earlier [141, 142], the finding by Evans *et al.* is the first to link such variants to *BRCA1* methylation status.

## *BRCA1* PROMOTER HYPERMETHYLATION MAY BE A CONSTITUTIONAL EVENT ARISING *IN UTERO*

Epigenetic gene silencing is a normal feature during embryonic development. Indeed, recent studies revealed that dramatic epigenetic alterations may occur already at the pre-implantation stage [143]. DNA methylation status varies between individuals and is influenced by genetic as well as environmental factors [48, 144–146]. The latter is particularly underlined by the fact that methylation patterns change with aging [147, 148], and that identical twins reveal much similarity at young age but grow more epigenetically different with time [144]. Assessing *BRCA1* promoter methylation in umbilical cord blood of >600 girls [138], we detected *BRCA1* methylation among 7% of them. Notably, the methylation profile across the CpG's mirrored the methylation status in healthy adults as well as ovarian cancer patients, indirectly supporting the hypothesis that *BRCA1* methylation is a constitutional event.

In order to be a risk factor for cancer, one may assume that methylation must persist over time. Taken together, our findings in newborns and adults are in accordance with the hypothesis that constitutional methylation may arise *in utero* and persist through life, constituting a cancer risk factor. Thus, such methylation follows a different pattern from methylation related to external influence and senescence [149] mirroring the difference between inherited subclonal mutations and hematological subclones carrying distinct gene mutations arising in response to accumulated genotoxic influence [150–153].

Consistent with our findings, Al-Moghrabi and colleagues, testing 300 newborns, found WBC *BRCA1* methylation in 9.9% of their cohort [154]. Moreover, they detected *MGMT* promoter methylation in 12.3% of newborns, revealing that neonatal methylation of tumor suppressor genes may not be restricted to *BRCA1* exclusively.

Interestingly, Al-Moghrabi and colleagues reported a potential association between *BRCA1* methylation status in mothers and their newborns [154]. While the data did not allow for formal statistical assessment, their explorative analysis indicated a moderate correlation, albeit not in accordance with Mendelian dominant inheritance [155]. Importantly, their findings do not define whether there was a paternal or maternal transfer of methylation. In some cases, transfer could be related to genetic variants (secondary epimutations) but in other cases it could be the transfer of primary epimutations. Considering germline mutations, mosaic mutations have been found related to neurological disorders [156], as well as in families with increased incidence of retinoblastoma [44, 45, 157]. In the latter case, mosaic mutations have been detected even as subclones in unaffected parents of an affected proband. This probably relates to such mutations arising somatically at the embryonic stage, and subsequently transferred through the gamete to the offspring. Further studies are needed to clarify this topic. Notably, among the patients with ovarian cancer and healthy controls that we examined, *BRCA1* methylation occurred independently of the two major haplotypes of the *BRCA1* promoter [138]. In line with lack of Mendelian inheritance patterns, this argues against a hypothesis suggesting constitutional methylation to be associated with a *cis*-acting factor.

The cause of *BRCA1* promoter methylation occurring among certain newborns is unknown. Yet there is substantial evidence linking prenatal factors to subsequent risk of different types of cancer in adult life, and breast cancer in particular [158–165]. As for methylation status in umbilical cord blood, global methylation patterns are associated with external factors like smoking during pregnancy, folate levels and famine [166–168], as well as birth weight [169]. To the best of our knowledge, studies evaluating the association between prenatal external factors and methylation of specific tumor suppressor promoters, such as for *BRCA1*, are lacking.

## POTENTIAL CAVEATS

While some studies applying genome-wide methylation analyses have detected differences in methylation of distinct CpG's related to incidental cancers [170–172], such differences in methylation in general occurred in CpG's located outside gene promoters; thus, the biological implications of these findings are uncertain. As for studies examining *BRCA1* promoter methylation with respect to breast and ovarian cancer, blood samples in general were collected from patients already diagnosed with their cancer. Thus, data assessing the predictive value of *BRCA1* promoter methylation to incidental cancers (by collecting blood samples years prior to diagnosis) are lacking. However, the risk of potential tumor DNA contamination, either from plasma free tumor DNA or circulating tumor cells seems negligible since the fraction of circulating tumor cells versus WBC detected in the circulation is estimated to be less than 1 to a million, and the concentration of free tumor DNA in the plasma is far lower than the DNA derived from WBCs [173–175].

On the other hand, distinct alterations in the WBC global gene methylation profile has been shown in patients with different cancers. This may not be directly linked to the cancer per se, but is probably related to alterations in WBC composition due to a cancer-related inflammatory response in patients with active disease [176–178]. This is consistent with the finding that WBC global gene methylation pattern varies between WBC subfractions [145, 179–182].

In our study on *BRCA1* methylation status and ovarian cancer risk, we performed extensive sensitivity analyses [138]. Here, we examined methylation status as a factor of tumor load, either by FIGO stage, or by examining methylation in patients who had recently had their tumors removed by surgery. Also, we examined methylation status in an additional cohort of ovarian cancer patients who had received chemotherapy. None of these factors influenced WBC *BRCA1* methylation status. Notably, we detected a methylation frequency which resembled that of healthy individuals (about 4%) across all subgroups of patients diagnosed with non-HGSOC, contrasting a methylation frequency of 9-10% among all subgroups of patients diagnosed with HGSOC.

Variations between WBC subfractions also need to be taken into consideration when comparing methylation among newborns versus adults. However, examining publicly available datasets [179, 180] we detected no variation in *BRCA1* promoter methylation patterns with respect to WBC subfractions, neither in newborns nor adults [138]. Thus, differences in WBC subfractions between cancer patients and controls is not a likely explanation why *BRCA1* methylation is increased among the patients diagnosed with HGSOC.

A final limitation relates to the use of conventional methylation-specific PCR (MSP) assessment methods, in as much as such methods do not allow for detailed quantification of the allele fraction being methylated. Neither do they inform whether cells are subject to mono-allelic or bi-allelic methylation. Such problems may be overcome by applying pyrosequencing [183] or contemporary next generation sequencing methodologies. This relates to mosaic methylation affecting a low allele fraction [138] in particular.

## WHAT ARE THE IMPLICATIONS OF THESE FINDINGS?

Merging evidence links constitutive methylation to cancer as well as other diseases, such as neurological disorders [184]. Further, we are beginning to learn how prenatal exposure (like smoking and diet) as well as maternal health issues may influence methylation status in the newborn [166, 167, 185, 186]. Most interestingly, experimental evidence has revealed acquired skills, like olfactory experience and sperm epigenetic programming in response to temperature, to be transmitted not only to the offspring, but into the third generation as well [187, 188]. Merging evidence indicates trans-generational responses also in humans [189, 190].

*BRCA1*, *MLH1*, *MSH2* and *MGMT* are all pivotal in DNA repair. With the exception of *MSH2*, all these genes are methylated in a significant fraction of certain cancer types. As for *MSH2*, we should recall the mechanism causing promoter methylation (deletion in the upstream Ep-CAM gene), making this mechanism unique in comparison to the others. As for other DNA repair genes for which WBC promoter methylation has not been linked to cancer, such as *BRCA2*, somatic methylation is a rare event in breast as well as ovarian cancer. Thus, it may well be that larger cohorts are needed in order to detect *BRCA2* methylation as a risk factor.

In colorectal cancers carrying *MLH1* tissue methylation, as well as breast and ovarian cancers carrying *BRCA1* methylation, a provoking question is whether these are acquired events occurring at some stage during tumor evolution, or if they may act as the primary event in the process of carcinogenesis. And in the latter case, could small groups of normal tissue cells that are methylated *in utero* act as cancer precursors? Importantly, mosaic germline mutations, likely to have occurred early during embryogenesis, have been detected in multiple genes related to different disease conditions in affected individuals (see [191] for additional details), including tumor suppressor genes like *TP53*, *RB1*, genes involved in neurofibromatosis type-1 and -2, the Fanconi syndrome as well as *BRCA1* [44, 156, 192–196].

Notably, DNA methylation status, similar to somatic mutations, continuously evolve during cancer progression [6, 197, 198]. Postulating *BRCA1*, *MGMT* or *MLH1* promoter methylation to be a “first event” in carcinogenesis in some individuals by no means exclude the possibility that it may occur as a secondary event at a later stage in other individuals. While pathogenic germline mutations of *TP53* in Li Fraumeni syndrome patients are likely to represent the initial event in breast cancers of such patients (as well as other neoplasia in these patients), recent studies revealed that somatic *TP53* mutations may arise at later stages of tumor evolution in many non-hereditary breast cancers [3, 4]. Accordingly, if a methylated tumor reveals a genomic signature mirroring the signature of a tumor arising in a germline mutation carrier with respect to secondary genomic events (*BRCA*-ness signature in breast cancers and MSI in colorectal cancers) the epigenetic event is likely to have occurred at a very early stage of tumor evolution. Alternatively, these tumors may have undergone selective sweeps [24] in response to an epigenetic event occurring at a later stage.

**Figure 3:**
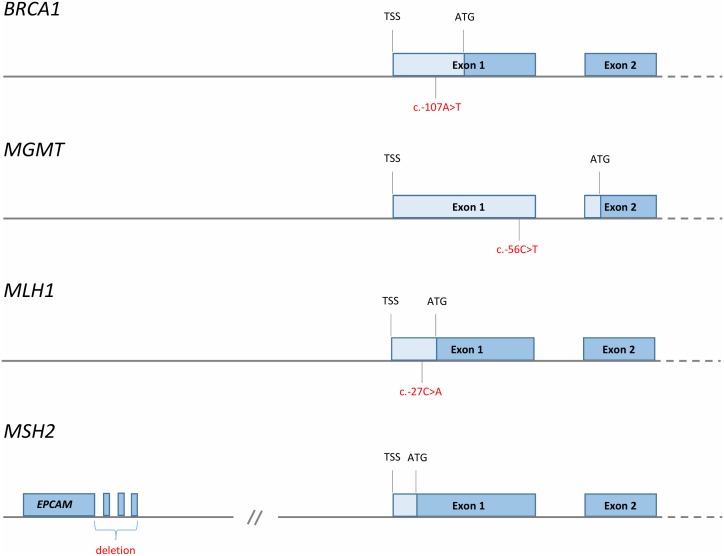
FIGURE 3: Reported *cis*-acting factors (red font) causing tumour suppressor promoter methylation and cancer risk [35, 43, 58, 133]. TSS; transcription start site, ATG; translational start site, blue boxes; protein coding regions, pale blue boxes; 5′UTR. x

Taken together, we believe there is substantial evidence indicating *cis*-acting mutations to be associated with promoter methylation in some cancer-prone families (secondary constitutive epimutations; **Figure 3**). In addition, the findings regarding partial *BRCA1* methylation in particular, but supported by similar findings for *MLH1* and *MGMT*, may indicate mosaic epimutations to be far more frequent than previously appreciated. Furthermore, such primary constitutive epimutations could contribute to a substantial number of cancer cases. The clinical implications of such findings, if confirmed, is substantial. There may be a rationale for offering routine testing of methylation status as part of general health control programs in adult healthy individuals. For instance, women carrying *BRCA1* WBC methylation could be offered regular ovarian surveillance by ultrasound exams from an age of 50 years. Secondly, the current findings should stimulate further research into the mechanisms by which such methylation arises, looking for potential pathogenic environmental influences or preventive strategies avoiding such events. While we lack selective drugs that may reverse gene-specific methylation as of today, such possibilities may become available in the future.

## Abbreviations:

HGSOC: – high-grade serous ovarian cancer,
HRD: – homologous repair deficiency,
LOH: – loss-of-heterozygosity,
MSI: – microsatellite-instability,
OR: – odds ratio,
PARP: – Poly-ADP-Ribose-Polymerase,
SNP: – single nucleotide polymorphism,
WBC: – white blood cell.
